# Advancing Targeted Protein Degradation via Multiomics Profiling and Artificial Intelligence

**DOI:** 10.1021/jacs.2c11098

**Published:** 2023-01-27

**Authors:** Miquel Duran-Frigola, Marko Cigler, Georg E. Winter

**Affiliations:** † CeMM Research Center for Molecular Medicine of the Austrian Academy of Sciences, 1090 Vienna, Austria; ‡ Ersilia Open Source Initiative, 28 Belgrave Road, CB1 3DE, Cambridge, United Kingdom

## Abstract

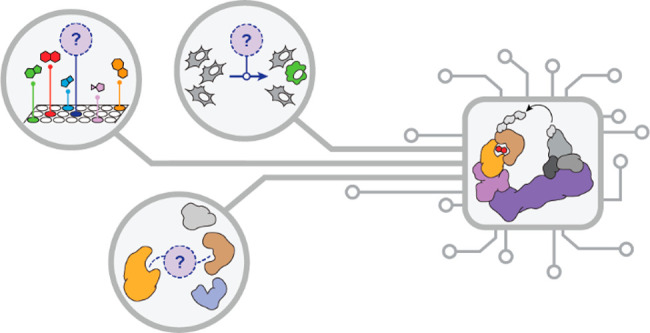

Only around 20% of the human proteome is considered to be druggable with small-molecule antagonists. This leaves some of the most compelling therapeutic targets outside the reach of ligand discovery. The concept of targeted protein degradation (TPD) promises to overcome some of these limitations. In brief, TPD is dependent on small molecules that induce the proximity between a protein of interest (POI) and an E3 ubiquitin ligase, causing ubiquitination and degradation of the POI. In this perspective, we want to reflect on current challenges in the field, and discuss how advances in multiomics profiling, artificial intelligence, and machine learning (AI/ML) will be vital in overcoming them. The presented roadmap is discussed in the context of small-molecule degraders but is equally applicable for other emerging proximity-inducing modalities.

Targeted protein degradation (TPD) is a pharmacologic modality that harbors the potential to address critical shortcomings of inhibitor-centric approaches. In order to deliver on these promises, it will be essential to continue to innovate and advance this field. In this perspective, we initially summarize how TPD is differentiated from small-molecule agonists and antagonists. Moreover, we are reflecting on some key challenges the field is currently facing, and how advances in multiomics profiling, artificial intelligence, and machine learning (AI/ML) can help researchers to address and overcome these challenges. In this context, we are reviewing strategies that have already been implemented, but also expand on approaches that should provide a tangible upside in the future.

## 1 Inhibitor-Centric versus Proximity-Inducing Pharmacology

### 1.1 Concepts and Limitations of Inhibitor-Centric Pharmacology

Most ligand discovery efforts are focused on small molecules that bind to hydrophobic pockets and aim to block the biochemical activity encoded by the bound site. Ligand optimization is focused on improving binding potency and residence time to maximize depth and duration of target occupancy to achieve therapeutic efficacy. Consequently, inhibitor-centric concepts of ligand discovery have recently been summarized as “occupancy-driven” small-molecule modalities.^1^ For many targets that are currently pursued in drug development, the occupancy-driven inhibitor discovery approach is in principle well suited. From a global perspective, these “low hanging fruit” aspects of ligand discovery however make up only around 20% of the human genome, thus leaving some of the most compelling targets outside the reach of small-molecule discovery approaches.^2,3^ This includes, among others, transcription factors or other intrinsically disordered proteins, as well as scaffolding proteins.^4,5^

Upon closer inspection, even a protein that is classified as ligandable can pose significant challenges to the drug discovery process. The ligandable domain might for instance be shared by a plethora of other proteins, thus complicating the development of selective inhibitors. Another issue could emerge from insufficient target characterization. Often, we lack functional resolution of a particular protein target, meaning that we do not necessarily know if the pharmacologically addressable domain is also relevant for the disease-association. For both scenarios, protein kinases can serve as a well-established example. The highly conserved ATP-binding site of the more than 500 kinases encoded in the human genome makes the discovery of selective inhibitors a challenging and an often unachievable venture.^6,7^ Moreover, it is also well-documented that many kinases feature scaffolding functions. This is best exemplified by the subfamily of pseudokinases, such as HER3. HER3 features very low catalytic activity but engages in oncogenic signaling by acting as a scaffold to enable receptor heterodimerization.^8−10^ Consequently, occupying the, *per se* druggable, ATP binding pocket of HER3 has been shown to be insufficient to functionally disrupt HER3.^11^ Importantly, even if a protein domain is not functionally compromised, benefits associated with small-molecule inhibitors might be limited. This is exemplified, among others, by bromodomain-containing proteins. The druggability of bromodomains was first established for the bromodomain and extra-terminal domain (BET) family of proteins.^12^ These results established the motivation for ligand discovery efforts for other bromodomain-containing proteins such as the histone-acetyltransferases CBP/EP300,^13−17^ the E3 ligase TRIM24,^18,19^ and the chromatin remodelers SMARCA2/4.^20^ While these efforts culminated in potent, specific, and well-characterized ligands for the individual bromodomains, ensuing functional analyses revealed that they are largely lacking cellular efficacy.^21^ In all cases, the targeted proteins also harbored multiple other functional domains and are often participating in intricate protein–protein interactions that appeared to be bromodomain independent. Collectively, these examples outline that the pharmacologically addressable domain is not necessarily functionally involved in the observed phenotype. In other words: a protein that appears “druggable” on the first glance might still not be within reach of the conventional occupancy-driven and inhibitor-centric pharmacology.

Lastly, even if a protein target can be meaningfully blocked by a small-molecule inhibitor, a potential therapeutic window can be compromised by toxicity that emerges from target inhibition in other tissues or cell types. This is a particular challenge if functional inhibition of the target requires a high fraction of target occupancy and inhibitors need to be dosed at high concentrations leading to elevated plasma concentrations.

Over the past decade, we have experienced tremendous progress in the innovation or further development of small-molecule modalities that overcome some of the limitations of occupancy-driven, competitive inhibitors. These innovations range from a renaissance of covalent small molecule inhibitors to advancements in proximity-inducing pharmacology, most notably the emerging field of TPD.^22,23^ In the following, we want to outline how TPD can address inherent challenges of occupancy-driven pharmacology. Next, we want to introduce three key challenges that TPD is currently facing and share our perspective on how these problems can be overcome. In particular, we argue that the advent of AI/ML technologies, coupled with multiomics profiling techniques for phenotypic screening, may pave the way toward efficient identification and rational design of small-molecule degraders.

### 1.2 Degraders are proximity inducers that can address inhibitor shortcomings

One avenue to overcome challenges of conventional inhibitors is to fundamentally change the perspective and perception of small-molecule action. An intriguing solution is to turn attention to small molecules that can change (rather than inhibit) protein function, allowing us to chemically rewire biological circuits. One way to change target protein function via small molecules is to induce naturally nonoccurring protein–protein interactions (PPIs) involving that target. This forms the conceptual foundation of chemically induced dimerization (CID), which is also referred to as proximity-inducing pharmacology.^24^ Some of the best-studied small-molecules such as rapamycin, FK506, or cyclosporin function via stabilizing non-natural protein–protein interactions.^25^ Collectively, these molecules have also been referred to as “molecular glues”. The concept of recruiting sterically confined effector proteins to inhibit target protein function is still actively pursued both in basic research and in translational studies. Due to space constraints, we want to refer to excellent recent reviews covering this space.^26,27^ The fundamental concept of leveraging proximity-inducing pharmacology to change biological circuits has been explored via a multitude of different avenues, also including biologicals.^28−31^ Small molecules, mostly of a heterobifunctional design, have been leveraged to install PPIs with kinases,^32^ phosphatases,^33^ acetyltransferases,^34^ and deubiquitinases,^35,36^ thus enabling researchers to alter posttranslational modifications on a target protein to change protein function. Moreover, incorporation of RNA-binding small-molecules into a heterobifunctional design has afforded ribonuclease targeting chimeras (RIBOTACs): small molecules that induce proximity between an RNA of interest and a ribonuclease to prompt RNA degradation.^37−39^

A powerful embodiment of proximity-inducing drugs are small-molecule degraders. Significant attention has been given to degraders that hijack the autophagy machinery;^40−42^ however, most efforts in the field focus on degraders that co-opt the ubiquitin-proteasome system (UPS).^43^ For the purpose of this perspective, we will hence exclusively focus on these compounds. Typically, they function by inducing the proximity between a target protein of interest (POI) and an E3 ubiquitin ligase. If the ligase and the POI are positioned in a favorable manner, the POI gets poly-ubiquitinated and degraded by the proteasome. Degraders thus harbor catalytic potential, meaning that one equivalent of degrader can induce the degradation of multiple POI equivalents. Due to this characteristic feature, degraders can achieve cellular potency that is far below their *K*_D_ for the respective POI. Most small-molecule degraders fall into one of two categories. On the one hand, heterobifunctional proteolysis-targeting chimeras (PROTACs) leverage two different ligands to simultaneously engage the POI and the ligase. Both ligands are connected by a flexible linker element that ensures proper spacing and architecture of the resulting ternary complex. On the other hand, molecular glue degraders (MGDs) connect ligase and POI by modulating protein surface topologies and orchestrating multiple PPIs between the involved proteins. They typically only bind one component in isolation, and ternary complex formation is driven by overall binding cooperativity. This enables the degradation of proteins that are generally considered to be unligandable targets for small-molecule modulation. A more in-depth differentiation between PROTACs and MGDs is discussed elsewhere.^44^

### 1.3 Small-molecule degraders can address challenges of occupancy-driven pharmacology

The pharmacology of degraders enables them to address many challenges that are inherent to the occupancy-driven drug design. Most importantly, degraders can extend the reach of the druggable proteome. Focusing on PROTACs, this is exemplified by degraders of the aforementioned bromodomain proteins SMARCA2/4, TRIM24, and CBP. When ineffectual bromodomain ligands were furnished into PROTAC targeting warheads, potent and selective degradation could be achieved.^45−47^ In sum, PROTACs hence allow ineffectual binders to be repurposed for conversion into functional degraders. PROTACs are therefore, in principle, able to address proteins that can be liganded, but fall short for proteins that are considered unligandable, such as the majority of transcription factors. Seminal work around thalidomide and its analogs (collectively referred to as “IMiDs”) has revealed a blueprint to close this gap. In brief, IMiDs bind the E3 ligase cereblon (CRBN), thus complementing a surface patch that subsequently recruits a variety of C2H2 zinc finger transcription factors for ubiquitination and degradation.^48−51^ Recruitment of these transcription factors (such as IKZF1 and IKZF3) happens in the absence of a measurable binding affinity between the IMiDs and IKZF1/3. Rather, IMiDs catalyze ternary complex formation by orchestrating multiple PPIs.^52,53^ Additional MGDs have been reported, which induce the degradation of proteins devoid of obvious ligand-binding pockets, such as RBM39^54−57^ or cyclin K (CycK).^58−60^ In sum, MGDs have shown the potential to induce the degradation of proteins that are considered unligandable. Interestingly, recent evidence suggests that MGDs depend on a certain baseline affinity between E3 and POI in the absence of the respective small molecule.^61^ Further research will be required to validate if this could be a predictive feature that can be exploited for *de novo* MGD design.

In addition to expanding the druggable space, degraders also address the challenge of inhibitor selectivity. There are multiple lines of evidence where promiscuous ligands could be furnished into highly selective PROTACs. Several mechanisms have been shown to contribute to this increase in selectivity. This includes steric incapability of some targets to form a productive ternary complex, or lack of a suitably positioned lysine residue for ubiquitination.^62−69^ Another layer of degrader selectivity has been observed between mutated and *wild type* target proteins. Among others, degraders with comparable affinity for both forms were shown to preferentially degrade BRAF^V600E^ over BRAF^WT^.^70^ In most reported cases, the observed selectivity of a particular degrader resulted from empirical optimization or was fortuitous, but first computational approaches empower rational design of selective degraders.^71^ Lastly, degraders might also be able to address challenges in dose-limiting on-target toxicity. For instance, it was shown that a PROTAC that co-opts the E3 ligase VHL to induce the degradation of BCL-X_L_ can spare the thrombocytopenia associated with systemic BCL-X_L_ inhibition.^72^ Mechanistically, this was rationalized by the low VHL levels in platelets. In addition, the catalytic mechanism of degraders can potentially omit the need for continuously high drug levels, thus providing another opportunity to limit toxicity. This advantage will be particularly evident for POIs with long half-life and slow protein resynthesis rates.

Collectively, small-molecule degraders offer solutions to address various limitations of occupancy-based inhibitor design. Arguably, many of these theoretical advantages and solutions need to be put into clinical practice. Pharmacokinetics (PK) and pharmacodynamics (PD) liabilities are likely to emerge for small-molecule degraders, especially in the case of the relatively large PROTAC compounds.^73^ Moreover, the field needs to evolve from episodically identified points of differentiation to a rational framework to design potent, selective degraders that address clinically meaningful challenges. In the following, we want to highlight some of the associated key challenges.

### 1.4 Current Challenges in the TPD Field

**Figure 1:**
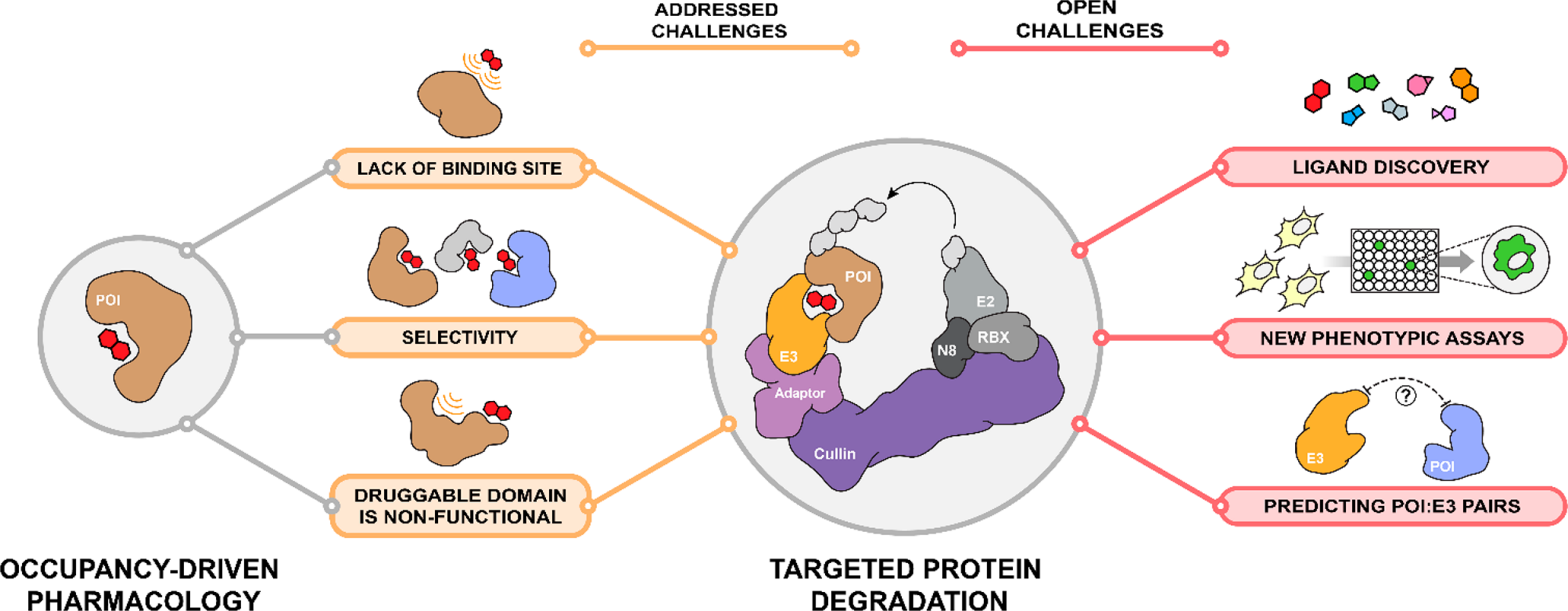
Addressed and open challenges of targeted protein degradation.

Many of the challenges of degrader modalities will be associated with translating preclinical data into effective medicines that provide measurable benefit in clinical studies. Given the scope of this perspective, we will here however focus on challenges that are associated with broadening the scope of degraders in a rational and data-driven manner. To this end, we want to focus our attention on three topics: (i) Expanding the reach of PROTACs through advanced ligand discovery efforts; (ii) Identification of MGDs via phenotypic screens coupled to cutting-edge target identification; (iii) Predicting compatible POI-ligase pairs for degrader discovery (Figure 1).

A key challenge (i) for PROTACs has been the dependency on a limited number of E3 ligases that can effectively be co-opted via potent and well-characterized ligands, preventing target- and cell-type selective degradation. Moreover, most reported targeting ligands are leveraging well-described chemical matter, such as kinase inhibitors. The dependency on existing ligands critically stymies progress toward undrugged proteins that are *per se* ligandable, but where no chemical matter has been reported. The discovery of MGDs has, for the most part, been serendipitous (ii). While first rational approaches have been reported,^59,74^ further scaling the discovery of MGDs will require innovations in phenotypic assays and in downstream target validation strategies. Finally, (iii) an important goal would be to develop a computational framework that can prioritize POI-ligase pairs that feature compatible surfaces, thus empowering the de novo design of degraders in a structure-informed manner.

## 2 Empowering Ligand Discovery with Proteomics and AI/ML

### 2.1 Proteomics-Empowered Ligand Discovery Approaches

The paucity of ligands for E3 ligases and currently undrugged proteins critically hampers progress in TPD. Traditional ligand discovery efforts typically focus on recombinant protein assays, aiming to identify small molecules that either bind a structured site or block the encoded biochemical activity. This includes, among many others, fluorescence polarization,^75,76^ surface plasmon resonance,^77^ thermal shift assays,^78,79^ and binding-enrichment assays that are amenable to DNA-encoded chemical libraries (DELs).^80^ These assays can inform ligand discovery when the assessed recombinant protein adopts a conformation that closely approximates the native fold found in a cellular environment. Unfortunately, many undrugged proteins do not fall in this category.

In contrast, chemoproteomics aims to map ligand–protein interaction on a proteome-wide scale, typically in intact cells. In principle, it couples a drug-affinity enrichment with mass spectrometry to identify proteins that bind to a given small molecule.^81−83^ A key advantage of this approach over conventional ligand discovery strategies is the ability to probe proteins in their native environments. This enables the discovery of actionable binding sites that might be undetectable or nonobvious in isolated, recombinant systems. For instance, chemoproteomics profiling has led to the discovery that the natural product nimbolide covalently ligands a cysteine residue in a disordered region of the E3 ligase RNF114. This enabled the design of RNF114-based degraders, also expanding to other chemotypes.^84,85^ Chemoproteomics is often employed to identify protein targets of phenotypic screening hits. Over the past decade, it has however also successfully been employed with fragment-like structures, aiming to prospectively identify specific fragment–protein interactions to enable ligand optimization. Initial work has predominantly focused on cysteine-targeting, covalent fragments.^86−88^ These approaches have the advantage that they not only inform on the liganded protein but also precisely reveal the covalently bound cysteine residue. Of relevance for TPD, this has recently identified fragments that covalently engage E3 ligases.^84,89−93^ Encouragingly, as mentioned above, these fragments could successfully be incorporated into functional PROTACs capable of inducing the degradation of BRD4 and other POIs. Future research will reveal if, in general, hits from covalent fragment screens can be advanced toward drug-like ligands for PROTAC or MGDs. Moreover, we will likely witness the continuation of a systematic expansion toward other amino acids including lysines or tyrosines.^94−97^ Of note, proteomics-enabled ligand discovery is not limited to covalent compounds. Equipping noncovalent fragments with a photoreactive diazirine moiety similarly enables their proteome-wide target identification without the prerequisite of a particular amino acid in or nearby a ligandable site.^98−100^ For both covalent and noncovalent compounds, the site of compound binding is typically revealed by introducing a cleavable connection between the fragment and the bead resin, or by using desthiobiotin.^88,101^ This step is instrumental to inform ensuing ligand optimization efforts. Below, we will outline how AI/ML-enabled docking studies can critically support this step, particularly for proteins that lack complete structural characterization.

In sum, proteomics-driven ligand discovery has the potential to significantly expand the reach of TPD and other proximity-inducing modalities. Ligands identified for undrugged POIs can be incorporated in a heterobifunctional PROTAC design. Identification of novel E3 ligands can empower further diversification of PROTACs. Elaboration of an E3-binding ligand into an E3-hijacking MGD is less obvious yet could be critically empowered by (protein–protein) docking^102,103^ and molecular surface interaction fingerprinting,^104^ as outlined in section 4 below. Next, we will discuss how purely computational methods can support ligand discovery, including identification of putative binding sites in protein structures, large-scale molecular docking, molecular dynamics simulations, and quantitative structure–activity relationship (QSAR) models.

### 2.2 Ligand Discovery Enabled and Empowered by AI/ML

**Figure 2:**
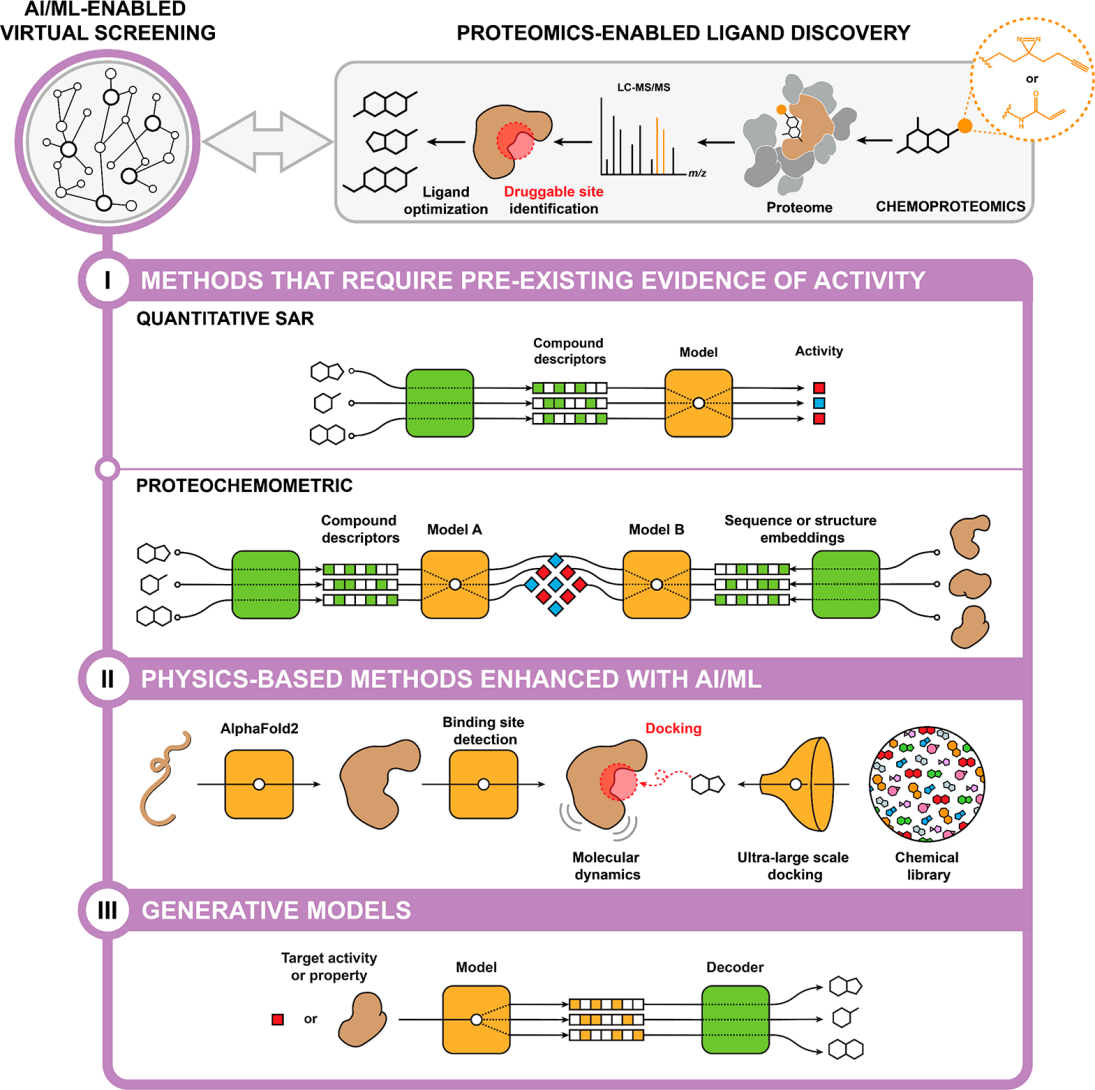
Advancing ligand discovery with proteomics and computational approaches. AI/ML-based methods can be divided into distinct categories (I–III) depending on the information they require as input.

Computational approaches to ligand discovery can be divided into two broad categories (Figure 2). On the one hand, there are methods that require pre-existing evidence of a certain activity (typically, binding affinity measurements for a target of interest). The classical representative of this category is QSAR modeling where structural/physicochemical properties of the small-molecules are correlated with experimentally measured values of activity. Based on the observed patterns of correlation, the activity of new compounds can be predicted. Translated to the field of AI/ML, QSAR can be seen as a “supervised” approach requiring training data. Indeed, a long repertoire of supervised AI/ML algorithms has been applied to the discovery of new ligand–protein pairs based on previous binding data,^105^ borrowing techniques from the fields of natural language processing,^106^ image recognition,^107^ and network analysis.^108^ In essence, AI/ML methods are able to dynamically engineer small-molecule features that are optimally correlated with the experimental outcome. Typically, AI/ML methods engineer molecular features based on a string (text) representation of the molecule (SMILES or, more recently, SELFIES^109^), or a small-molecule graph where nodes are atoms and edges are bonds annotated with their respective properties (atomic weight, valence, etc.). Modern supervised AI/ML algorithms have shown outstanding performance in a broad set of biophysical and physiological prediction tasks, and are now incorporated in QSAR benchmarks.^110^ In TPD, QSAR models have for instance been employed to identify novel glutarimide analogs that promote selective degradation of IKZF3 and/or GSPT1 via CRBN.^111^

A fundamental limitation of supervised AI/ML is the requirement of previously available experimental data. This is aggravated by the fact that advanced AI/ML methods are generally data-greedy, meaning they require more data than the classical set of QSAR techniques. As a result, supervised AI/ML for ligand discovery is best suited for well-studied ligandable proteins. Indeed, recent efforts have broadened the applicability of supervised AI/ML models to low-data and no-data scenarios,^112,113^ i.e. to under-studied and unliganded proteins. Conceptually, the improvement has been to simultaneously train over millions of experimental data points (spanning thousands of targets) contained within large chemogenomics databases such as ChEMBL,^114^ BindingDB,^115^ or PubChem BioAssay.^116^ This kind of global training is laid out as a “bimodal” AI/ML model, where both the ligand structure and the protein sequence/structure are passed as inputs. Thus, the so-called proteochemometric methods focus their attention not only on the properties of the ligands but also on the proteins, and ultimately aim to discover the rules of protein–ligand recognition. In this context, algorithms to embed protein sequences into dense numerical representations that are amenable for AI/ML offer promising opportunities. These numerical representations have been proven to capture a wide spectrum of protein characteristics, including structural domains, family membership, and function.^117−119^ One advantage of proteochemometric approaches, compared to the classical “one protein, one model” rationale, is that, at least in principle, ligand–protein interaction predictions can be done for any protein of interest, as long as their sequence is available. However, the applicability of existing proteochemometric methods is still limited to relatively well-studied regions of the protein space, enriched with ligandable proteins for which a substantial amount of experimental data is available. In practice, the performance of proteochemometric methods may vary depending on protein families, with a bias toward protein families that are already considered to be ligandable.^120^ Extrapolation to genuinely novel targets, belonging to unliganded regions of the sequence space, is a major challenge for current supervised AI/ML methods. In this line, passing chemoproteomics data through these models may increase their domain of applicability,^121^ since these high-throughput assays are likely to introduce new evidence for unseen targets and target families.

On the other hand, there are computational approaches that do not require previous knowledge of binding affinity (Figure 2). Traditionally, these methods have relied on physics-based calculations obtained through docking procedures and/or molecular dynamics simulations. An advantage of physics-based methods is that they are, in principle, applicable to any protein of interest as long as a high-resolution 3D structure is available. For years, the need for 3D structural data of the proteins has been perceived as a critical limitation, especially for membrane-bound proteins which are difficult to crystallize.^122^ Ligand docking has been used with homology models of protein structures,^123^ although the applicability is limited by the need for structural templates from close homologues,^124−126^ since atomistic details in the binding site are crucial determinants of affinity. However, recent breakthroughs in proteome-wide AI/ML-based structure prediction solely based on sequence may help overcome this limitation. The unprecedented accuracy of AlphaFold2,^127^ RosettaFold,^128^ and others^129,130^ offers, at least in principle, the opportunity to virtually screen any protein of interest with the structure-based approach, irrespective of its *a priori* ligandability (after AlphaFold2, the structural coverage of the human proteome increased from 48% to 76% of the total residues).^131^ While there is ongoing debate as to whether e.g. AlphaFold2 structure predictions are of sufficient quality for protein–ligand docking,^132,133^ it is clear that the possibility is within reach, even if some filters and extra preparation steps like molecular dynamics relaxation may be required.^134^ In combination with knowledge gathered from chemoproteomics experiments, such as mapping of the ligand binding site, the accuracy and speed of structure-based simulations could be boosted, providing a tool to quickly screen compounds against fully structurally resolved proteomes.

Identification of putative binding sites in a protein structure is a first, advisable step in docking procedures. Narrowing the search space to specific regions of the protein allows for higher throughput compared to “blind” docking. Computationally, binding pockets can be identified through inspection of geometrical features in the protein surface, presence of putative hydrogen bond donors and acceptors, spots of hydrophobic contact, etc.^135−137^ AI/ML models have been applied to the identification of these potential pockets, and especially to the up-ranking of the most ligandable ones.^138^ Such predictions will be highly complementary to ligandability data obtained from chemoproteomics experiments. Following binding site identification (either fully computationally or supported by wet-lab evidence), each candidate ligand can be evaluated in the site region, where multiple binding poses are tried and scored. Evaluation can be done in flexible mode, allowing for induced fit between the ligand and the protein. Assessment of covalent binding is also possible,^139−141^ which may be relevant to complement certain chemoproteomics screening platforms.^23^ Recently, enhanced docking pipelines have been proposed to drastically improve the computational feasibility of docking procedures, enabling the screening of ultralarge “make-on-demand” chemical libraries such as the Enamine REAL collection.^122,142,143^ For example, a 100-fold acceleration of the process was observed by docking a fraction of the chemical library in synchrony with an AI/ML ligand-based prediction of the remaining docking scores.^144^

### 2.3 Ligand Library Design Aided by AI/ML

Coupled with the augmented virtual screening capacity, we are witnessing an avalanche of “generative” AI/ML models capable of designing new small-molecule structures with desired target properties (Figure 2).^145−148^ Generative models have been used to compile both focused and diversity-oriented chemical libraries,^149,150^ to design inhibitors *de novo*,^151^ and to perform hit-to-lead optimization,^152^ typically in a multiobjective mode where e.g. selectivity, synthetic accessibility, and drug-like properties are improved simultaneously.^153,154^ Of note, these algorithms can be interfaced with docking calculations in order to design small molecules with high docking scores.^155,156^ This could simplify the design of compounds binding to unliganded proteins. Mature software tools now exist to run generative models on conventional computers,^154,157^ and community-accepted benchmarks enable the systematic assessment of generated small-molecules, considering not only their predicted activity but also their novelty, validity, and diversity.^158,159^

Progressively, and despite initial limitations and criticisms,^160^ AI/ML-based generative models are being incorporated into realistic experimental settings. For example, by constraining compounds with a subset of simple reactions and building blocks, a “synthesis-on-a-chip” procedure was devised where the AI/ML tool suggested compounds that could be immediately synthesized.^161^ Other specialized methods focus on fragment elaboration,^162^ which could be employed downstream of proteome-wide fragment screening experiments via aforementioned chemoproteomics. Moreover, existing platforms have been versioned to design optimal linkers connecting the two molecular subunits in a PROTAC.^163^ Design of PPI inhibitors has also been attempted,^164^ which may set the stage for the design of MGDs. As more degraders are discovered, it is likely that dedicated generative models will flourish, borrowing principles apprehended in generalistic models^165^ and fine-tuning them to the degrader-specific tasks. Adaptability to a chemical space of interest by adjusting the inner parameters of a given model is a unique feature offered by AI/ML.

## 3 Molecular Glue Degrader Discovery via Advanced Phenotypic Screens

### 3.1 Phenotypic Discovery of MDGs

Historically, cell-based phenotypic assays have provided the richest source for MGD identification. The clinical evaluation of lenalidomide, one of the aforementioned CRBN-modulating IMiDs, was motivated by phenotypic assays for caspase cleavage of thalidomide analogs.^166^ Similarly, a straightforward screen for IMiD-induced cytotoxicity in cells with low CRBN levels revealed the highly potent IMiD CC-92480,^167^ which has recently entered clinical investigation. Here, the rationale for screening in CRBN low cells was to select for compounds with high potency and catalytic turnover that could overcome limited availability of CRBN. The discovery of CC-885 as a CRBN-dependent GSPT1 degrader^53^ was likewise initially motivated by cancer cell line profiling studies, prompting the development of the clinical candidate CC-90009.^168,169^ Cell-viability-based phenotypic assays have also led to the identification of several MGDs that induce the degradation of CycK. One study reported a strategy based on chemical profiling in cells deficient for UBE2M.^59^ As UBE2M is required for the activity of a large fraction of all 250 cullin-RING ligases (CRL), this screen was poised to identify small molecules that functionally depend on a CRL. Another study analyzed correlative data to identify compounds whose activity correlates with expression of the CRL adaptor protein DDB1.^58^ Also this study led to the identification of chemical matter that induces CycK degradation. Both studies converge on a shared mechanism of action where the identified compounds directly glue the CDK12-CycK complex to DDB1, thus prompting CycK degradation in the absence of a dedicated substrate receptor. Collectively, these examples demonstrate the relevance of cell-based assays as a starting point for MGD discovery. However, while increasingly more elaborate in their design, these studies fundamentally still relied on cell-viability-associated readouts. This comes with the limitation that the scope of protein targets is limited to those with an essential function in cell viability/cellular fitness. In this section we want to discuss how advanced phenotypic screens will enable the identification of next-generation MGDs, and how innovations in phenotypic readouts will require matched advancements in target identification strategies. In the following, we discuss how richer phenotypic readouts can contribute to a better understanding of TPD. We also present emerging AI/ML techniques that can contribute to efficient processing and integration of these complex data sets. In sum, we hypothesize that these advancements will be necessary in order to expand the reach of phenotypic assays to protein targets that are not essential for cellular fitness.

### 3.2 Introducing Diversity in Phenotypic Screens

Conceptually, we anticipate that advancements in phenotypic screens will emerge from introducing diversity that can be measured, quantified, interpreted, and predicted. Diversity can emerge from different genetic backgrounds, cell types, metabolic states, environmental conditions, or exposure to perturbagens, among others. In many examples that are mentioned in the following, this necessitates implementation of automated microscopy workflows that empower readouts on a single-cell level. Alternative methods are, for instance, based on screening approaches with a single-cell transcriptomic readout.^170^ Segregation of different cell types/states in microscopy images can be conducted by staining,^171^ by in situ sequencing,^172^ or by segregation based on morphological features which is often enabled by AI/ML.^173,174^

One strategy to introduce diversity is to conduct phenotypic screens not in homogeneous populations of cancer cell lines, but instead in a cellular background with more physiological relevance. To that end, several examples have documented the power of chemical screens in organoids^175,176^ or directly in patient material.^177−179^ Another avenue to introduce heterogeneity is to genetically engineer cells in a pooled fashion. To identify novel degraders, a selection of several E3s could for instance be inactivated via pooled sgRNA transduction (one E3 ligase knockout per cell). The (degrader) drug-induced phenotype of interest, for instance cell differentiation, could thus be quantified as a function of E3 ligase activity. In other words, this approach would allow the identification of drugs that depend on a particular E3 to induce a phenotypic/morphologic change on a cellular level. Another strategy to introduce complexity is to endogenously tag libraries of POIs, for instance with a fluorescent protein or a self-labeling tag.^180,181^ Again, this would enable quantification of drug effects on abundance or localization of multiple POIs in a pooled format. Identification of the individual clones can again be performed retrospectively, either by morphologic features specific to a particular clone (EGFP-POI localization and intensity) or via in situ sequencing. Recognition can further be advanced, for instance via weakly supervised deep learning.

### 3.3 Maximizing the Depth of Downstream Analysis

In addition to increasing the diversity of the screened cell populations, another strategy to exploit phenotypic screening is to maximize the depth of the downstream analysis. Linked to advanced data integration techniques, phenotypic readouts can provide functional insights about the compound, highlighting engaged cellular processes (e.g., degradation pathways) and dominant mechanisms of action. For example, the CDK inhibitor CR8 was identified as a CycK MGD by correlating gene expression levels of the E3 ligase adaptor DDB1 with cell sensitivity data.^58^ AI/ML may unveil more complex connections between molecular and phenotypic events. Ideker and co-workers showed that AI/ML network architectures can be crafted following the logic of a hierarchy (ontology) that reflects the known machinery of cells.^182,183^ In this network, inputs are genes, and calculations are forwarded from the more specific terms (e.g., protein complexes) to the more general ones (e.g., organelles or broad terms such as DNA repair), traversing pathways and biological processes. Applied to a genotype-phenotype association study of yeast genetic screening data, it was shown that the AI/ML network could map mutated genes to cellular growth rates.^184^ More importantly, inner, specific subsystems were highlighted in the mapping, reflecting the cellular subsystems activated in response to the genetic perturbation. Similar strategies have been applied in the context of prostate cancer clinical data.^185^ It is expected that more informative readouts, such as the aforementioned high-content microscopy data, will allow this kind of “visible networks” to interpret cellular responses beyond growth and proliferation. In turn, this will provide more detailed insights about the processes that connect a small-molecule perturbation to an observed phenotype. In this context, identification of degraders could be enabled by searching for compounds that trigger a cellular response that is functionally linked to the ubiquitin-proteasome system or any other protein degradation pathway. Indeed, PROTAC phenotypic signatures have already been identified through cell morphological profiling.^186^

### 3.4 Preparing Data for AI/ML-Enabled Analysis of Phenotypic Screening Data

**Figure 3:**
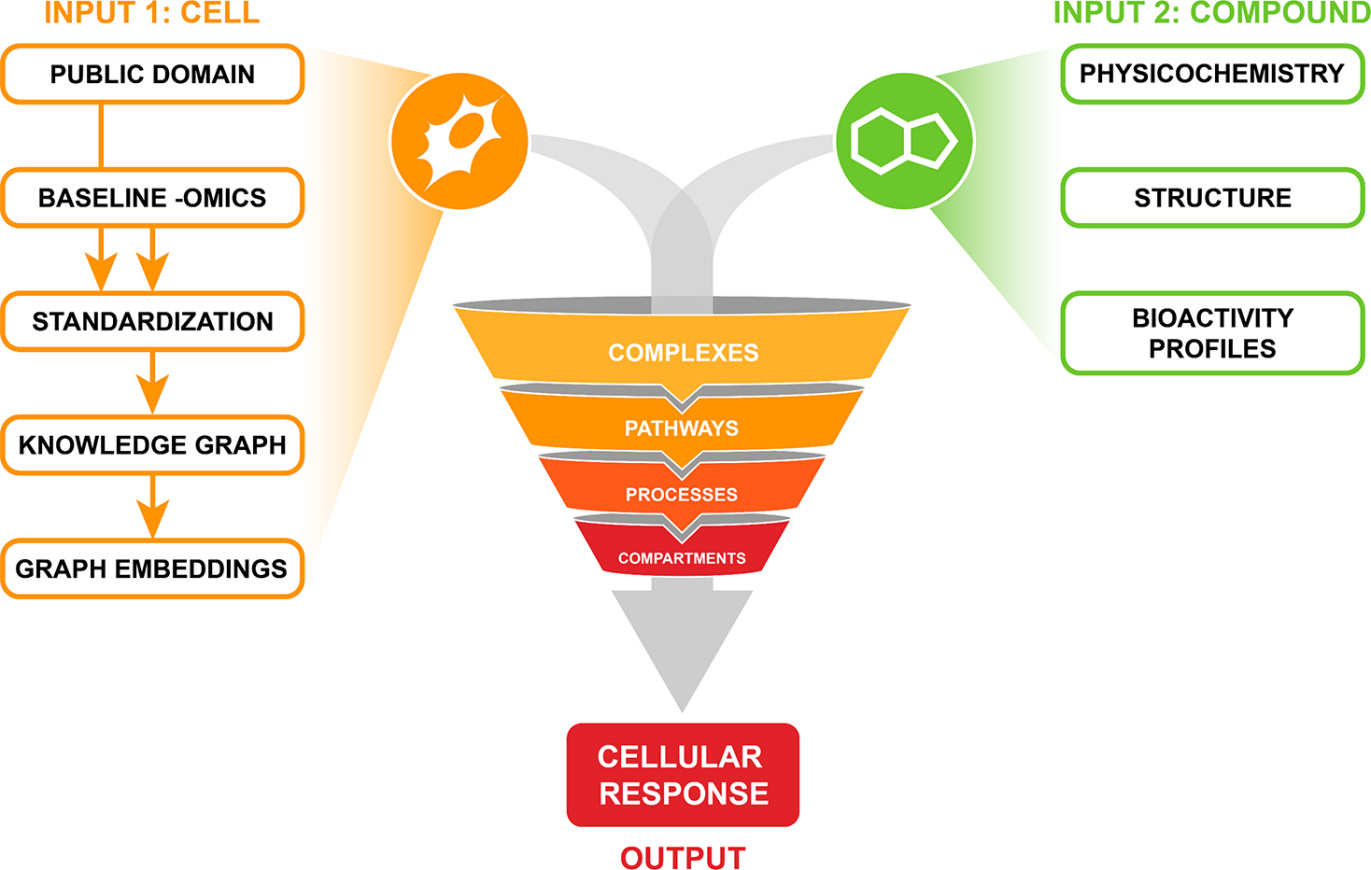
Preparing phenotypic screening data for AI/ML. AI/ML models enable the analysis of phenotypic screening data by translating cell- and compound-based features (input) into cellular response (output).

At a high level, an AI/ML model based on phenotypic screening data will have two inputs (e.g., a cell and a small-molecule) and one output (i.e., a cellular response) (Figure 3). Pharmacogenomics cancer cell-line panels fit into this scheme. Cancer cell-line collections are deeply annotated, offering a wealth of data to the “cell” branch of the input, including mutational, gene expression, protein abundance, and epigenetic profiles, among others.^187,188^ All of these -omics profiles express the baseline status of the cell, and are thus preperturbation data that can be passed to the AI/ML model either independently or in multiplexed form.^189−191^ Likewise, a broad set of traditional and modern small-molecule descriptors are able to capture physicochemical properties, 2D/3D structural information, and even *a priori* knowledge of compound bioactivities,^192,193^ which means that, on the “small-molecule” branch of the input, there is also a large amount of information available. Converting raw -omics and molecular data into an AI/ML-friendly form is a challenging task due to the high dimensionality of the data sets, biases, and artifacts specific to the -omics technique, and inconsistencies in units and identifiers. The challenges are exacerbated when input data are collected from multiple sources in the public domain. To ease this task, large biological knowledge graphs have been suggested as an intuitive framework for data integration,^194−196^ followed by graph embedding techniques capable of expressing the context of each entity (e.g., a compound or a gene) in a dense vectorial format. In brief, vector “embeddings” capture the interactions and statistical correlations found in the knowledge graph, such that highly connected nodes will have similar vector embeddings. With this procedure, heterogeneous large-scale data sets (e.g., compound-protein or gene-cell associations) can be formatted in a unified manner, and can be naturally compared or merged in multilayered analyses.^197,198^ It has been shown that embedding techniques can reduce the dimensionality of the input data by several orders of magnitude without affecting the predictive capacity of downstream AI/ML models.^196^ Moreover, the complexity of the downstream analysis is reduced substantially—in a recent analysis of a deubiquitinating enzymes (DUBs) genetic screen, DUB inhibitors could be identified based on simple similarity measures between embedded public data of drugs and the DUBs of interest.^193^

### 3.5 Processing Rich Phenotypic Readouts

Despite the rich annotation of small molecules and unperturbed cell lines, most pharmacogenomics panels have a very simple readout, typically a growth inhibition measure. This is reflected as a single-valued data point in the output branch of the AI/ML model. Even with such a simple readout, genes and pathways that determine drug response can be identified,^199,200^ and AI/ML models can be pretrained and then fine-tuned with organoids or patient data to achieve clinically relevant predictions.^201^ We anticipate that these notions will be adapted to the TPD field. By analyzing growth inhibition profiles across cell lines, it was for instance found that the drug efflux pump MDR1 confers resistance to PROTACs.^202^

**Figure 4:**
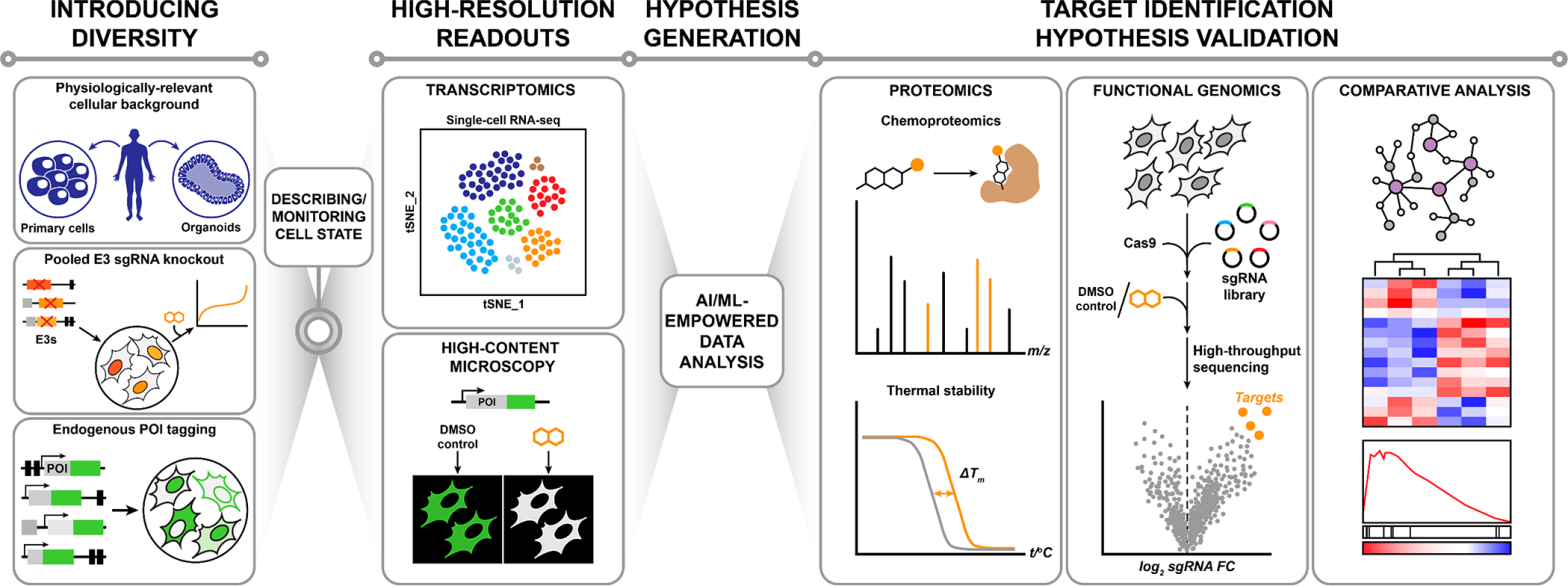
Schematic outline for advanced phenotypic screens. We envision that the innovation of the phenotypic discovery of MGDs will emerge from integrating changes in experimental screening approaches (through diversifying screened cell populations, implementing high-resolution single-cell readouts and further development of compatible target identification methods) with AI/ML-driven data interpretation.

Phenotypic screens are increasingly delivering richer outputs, including high-content microscopy readouts and postperturbation transcriptomics measures such as bulk- or single-cell RNA-seq (Figure 4). The goal of these data-rich readouts is to capture in more detail the cellular response triggered by a chemical compound. For example, observed changes in cellular morphology can help categorize compounds in broad mechanistic categories (e.g., alteration of intracellular metabolism, DNA transport, etc.)^203^ and differential gene expression profiles analyzed with conventional bioinformatics techniques can reveal dysregulated cellular pathways.^204^ Of note, the LINCS consortium, and in particular the L1000 Connectivity Map resource, provides a catalog of postperturbation transcriptomics profiles for thousands of compounds, offering a global map of the phenotypic response landscape of the medicinal chemistry space.^205^ The common way of exploiting this resource is through “connectivity” analysis, i.e. by comparing gene expression signatures of interest to a reference set of signatures of well-annotated compounds.^206^ It has been shown that compounds eliciting similar transcriptional signatures tend to share mechanisms of action.^170,207−209^ A recognized limitation of the approach is the confounding effect of transcriptional patterns attributable to the cell line, which complicates the extraction of compound-specific effects. Advanced transcriptional similarity metrics derived through AI/ML approaches can help ameliorate this bias.^210^

In addition, the LINCS consortium is performing genetic shRNA, overexpression, and CRISPR screens. Mechanisms of action can be revealed by phenocopying compound transcriptional readouts and these genetic profiles. In this case, similarity measures can be confounded by intrinsic differences between small-molecule and genetic perturbations, which further justifies the need for AI/ML “metric learning” approaches. For the specific purpose of degrader discovery, these differences are less essential as pharmacologic induction of POI degradation often phenocopies the genetic perturbation via shRNA or sgRNA. Recent genome-wide CRISPR screens with a single cell RNA-seq readout are providing vast collections of transcriptional phenotypes,^211^ with a clearly identifiable protein degradation cluster that can be used as a reference for small-molecule degrader discovery. Likewise, comparison of high-content imaging outcomes (either in their raw image forms or as extracted geometric features) can be framed as a “metric learning” problem for which AI/ML is ideal. Recent efforts to map the phenotypic landscape of genetic perturbations^212,213^ will provide a reference data set of imaging data to which compound phenotypic readouts can be matched and compared.

While similarity-based interpretation of transcriptomics and imaging profiles is likely to remain the by-default analytical framework for phenotypic screens, recent AI/ML-based approaches point toward standalone or direct mechanistic interpretation of phenotypic readouts. In particular, functional ontologies have been mapped to microscopy images,^214^ and mechanistic models of transcriptional readouts are becoming established tools.^215^ In summary, we believe that the necessary computational elements are already in-place for maximized, in-depth interpretation of phenotypic readouts. We anticipate that future degraders will frequently be discovered by phenotypic assays able to illuminate corners of the biological space that have not yet been explored with small-molecule perturbations.

### 3.6 Associated Requirements for Target Identification Approaches

Advanced phenotypic assays will also come with a need for experimental target identification (ID) approaches. In accordance with the increased resolution of phenotypic screens, target ID will similarly need to be compatible with single-cell formats. These might involve single-cell proteomics profiling,^216−218^ spatial metabolomics,^219,220^ or CRISPR/Cas9 screens with a scRNAseq readout.^211,221−223^ Conceptually, we differentiate between target ID methods that aim to (i) identify the drug-bound protein target, strategies that (ii) functionally assign effector proteins to drug action, and methods that (iii) holistically describe drug impact in an unbiased manner and aim to develop a mechanistic hypothesis based on comparative analysis with existing, large scale databases (as outlined in section 3.5) (Figure 4).

Approaches to identify the target protein for a small molecule are, largely speaking, dominated by proteomics. Aforementioned chemoproteomics approaches are the gold-standard to identify protein target(s) bound by a given small-molecule hit of interest. A prototypic example is the identification of CRBN as the protein target of thalidomide, which was enabled by a chemoproteomics strategy that employed a tethered thalidomide analogue.^48^ A potential downside of this approach is the necessity to chemically derivatize the compound of interest to allow tethering for ensuing affinity enrichments. In most cases, attachment points for an immobilization strategy might not be obvious. In other cases, the mode of molecular recognition of a compound might simply not allow any major derivatization. In such scenarios, orthogonal proteomics applications have emerged that are based on the notion that ligand binding (i) thermally stabilizes the target protein or (ii) protects a protein from proteolysis. Thermal stabilization (i) is leveraged in thermal proteomics profiling (TPP), an approach that has been employed to, for instance, identify the ferrochelatase FECH as a frequent off-target of kinase inhibitors,^224^ or to uncover leucine aminopeptidase 3 as an off-target of several HDAC inhibitors.^225^ The fact that ligand binding can protect from proteolysis forms the foundation of the limited proteolysis approach (LiP). Among others, this approach has identified the mechanism of action of a fungicide as a kinase inhibitor.^121^ Particularly focusing on target identification for small-molecule degraders, proteomics has been key to identifying degraded targets.^226^ In addition to global expression proteomics that assess changes in protein abundance after drug treatment, other approaches enable a more in-depth analysis of drug impact on nascent and mature proteins which allows approximation of drug effects on protein stability.^224,227,228^ As of now, none of these target-ID focused proteomics approaches have been shown to be compatible with single-cell analysis. As a consequence, we lack the resolution to describe small-molecule interactomes or drug impact on the proteome at a level of granularity that matches transcriptomics approaches. However, recent traction in the field of single-cell proteomics promises to overcome these hurdles in the near future.^229−231^ Target identification and elucidation of the mechanism of action of degraders via functional genomics was initially mostly driven by pooled CRISPR/Cas9 screens with a positive selection readout based on cellular fitness (“drug resistance screens”).^168,232,233^ Future readouts will increasingly focus on readouts that address POI abundance, or states of posttranslational POI modifications, such as (poly-) ubiquitination.^234,235^ Of note, a recent breakthrough in high-speed fluorescence image-enabled cell sorting now opens up possibilities to functionally assess effects of gene knockouts on POI localization.^236^ For the purpose of drug target identification, this strategy can be used to map all genes that are functionally required for a drug-induced change in POI localization such as nuclear export of a transcription factor. On another level, future target identification approaches will aim to increase the resolution of functional genomics from the protein level to the level of individual domains and amino acids. To this goal, base-editor screens, deep mutational scanning (DMS), and CRISPR tiling screens can play an important role. For instance, both DMS and CRISPR tiling have recently highlighted hotspots in ligase and POI that are functionally relevant for degrader activity.^237,238^

## 4 Enabling Prospective Degrader Discovery

### 4.1 Leveraging Low-Affinity Protein–Protein Interactions

In parallel to phenotypic screening, rational design of small-molecule degraders is likely to be enabled by recent progress in AI/ML. As explained in section 2.3, *de novo* design and hit-to-lead optimization are possible through generative models, which are constrained or guided by a given task. In TPD, the task involves the assembly of a ternary complex consisting of the POI, the E3 ligase, and the PROTAC or MGD.

It has been suggested that baseline compatibility between the POI and the E3 is a necessary requirement for the formation of a ternary complex.^61,239^ This implies that existing docking methods to discover PPIs and resolve interaction interfaces are likely to play a key role in the modeling of TPD mechanisms. However, there are several considerations to be made before attempting out-of-the-box application of docking in this scenario. First, the POI and E3 ligase are generally not cognate interaction partners. As a result, binding interfaces will likely be transient with weak affinity. Accurate modeling of low-affinity interactions is a recognized challenge for docking methods.^240^ Moreover, widely used and computationally efficient template-based docking methods are probably not applicable in TPD, since aid from structural alignment to known homologues may not be available.^241^ In general, ab initio docking methods will be more appropriate. One advantage of ab initio docking is that it allows for discriminating cognate and noninteracting protein pairs without the need for pre-existing evidence of a PPI. Another advantage is that it capitalizes primarily on physicochemical and shape complementary calculations, which does not impose constraints on known interologs. A major disadvantage is that the search space of docking poses can be unattainable computationally. For this reason, strong assumptions such as rigidity of the monomers,^242^ or stepwise pipelines starting with the identification of putative binding sites in each monomer independently,^243^ are often necessary to increase the feasibility of the approach.

Ultimately, in the context of TPD, the setup of a large-scale computational prediction would consist, on one side, of structures of the E3 ligases and, on the other side, of a set of POIs to be matched to the ligases. The first task would be to predict whether the POI-E3 PPI can potentially occur, and the second would be to provide a structural model for the interaction. Ab initio docking may naturally encompass both tasks, as demonstrated by recent approaches focused on PROTAC discovery.^244^ Additionally, data-driven methods may help discard unfeasible POI-E3 pairs early in the pipeline. For example, methods to assess the PROTACtability of POIs have been suggested,^245^ prioritizing over a thousand proteins with potential for becoming PROTAC targets. More generally, many PPI prediction methods exist, most of them based on protein sequences.^246^ The current state-of-the-art in sequence-based modeling is the use of AI/ML embeddings pretrained on multimillion-scale protein collections.^118^ These embeddings can capture evolutionary traits of the proteins, as well as functional and structural characteristics.^119^ As such, contrary to more complicated data structures such as phylogenetic trees or sequence alignments, they can be used directly as numerical inputs for predictive tasks, and to uncover relationships between proteins through straightforward distance calculations. Moreover, at least two follow-up applications of AlphaFold2 suggest that AI/ML may play an important role in the structural modeling of POI-E3 pairs. First, PPIs have already been modeled with small adaptations to the original algorithm.^247,248^ Second, protein design models have shown that new folds can be successfully predicted beyond the structural landscape explored by nature.^249^ Applied to POI-E3 ligase pairs, this suggests that existence of coevolving residue pairs at the PPI interface may not be a requirement for accurate modeling of structural contacts.

### 4.2 Inclusion of the Small-Molecule Degrader Early in the Modeling Pipeline

**Figure 5:**
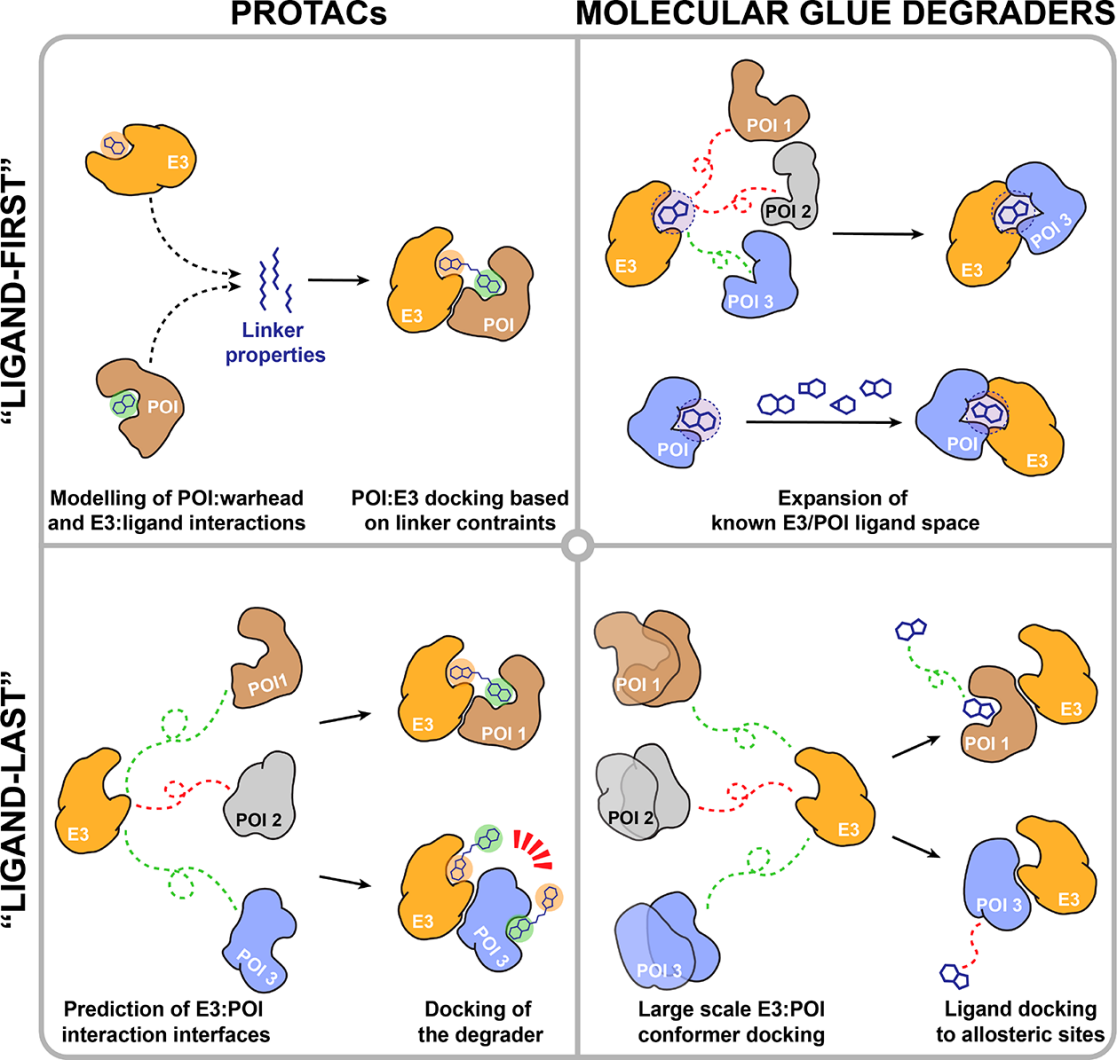
Computational approaches to prospective degrader discovery.

One obvious limitation of considering the POI-E3 ligase pair in the absence of the degrader is the fact that structural conformations induced or stabilized by the ligand are not taken into account. While structural modeling with apo (unbound) forms of the POI and E3 monomers may be sufficient in some ternary PROTAC complexes, it is generally recognized that the ligand plays an important role in determining the PPI interface, either by reshaping it to increase affinity (as it happens in every MGD) or by imposing occupancy and distance constraints (as in the case of PROTACs). Thus, several TPD ternary complex modeling approaches favor the use of holo (bound) forms. Indeed, the holo form of CRBN was used in a large-scale docking run to predict C2H2 zinc-finger degrons.^51^ Similarly, the computational pipeline PRosettaC starts by modeling POI-warhead and E3-recruitment ligand interactions, and then exploits the fact that PROTAC linkers have length constraints to drastically reduce the docking search space (Figure 5).^71^ In this line, efficient optimization techniques have been proposed to sample the space of ternary complex candidates using composite scores that take into account both PPI docking results and a PROTAC score that measures the feasibility of accommodating the ligand given the position of the binding pockets.^250^ Additional biological constraints can be incorporated to further restrict the search space, such as the location of the lysine in the POI for ubiquitination, or of known residue mutations that modulate complex formation. Overall, though, given the abundance of rotatable bonds in the small-molecule degrader, and the importance of protein backbone flexibility in the coordinated formation of PROTAC ternary complexes, modeling these assemblies is likely to remain a computationally intensive task despite the constraints imposed by PROTAC-bridged complexes. The small number of experimentally resolved ternary complexes further complicates benchmarking of methods.

### 4.3 Toward Computational Pipelines for MGD Discovery

All of the challenges above are enhanced in the case of MGDs. Currently, there is no well-established computational framework to predict and evaluate compound-induced PPIs.^251^ Therefore, the discussion presented below refers to potential, envisaged approaches, rather than a compendium of validated methods.

As a result of the scarcity of rational design guidelines, serendipity plays an important role in the discovery of MGDs.^252^ Progressively, the chemical characteristics that make a good MGD are being identified, such as addition of solvent-facing functional groups in already known binders.^25,26,253^ In addition, systematic screening efforts have revealed high glue hit rates in drug-like collections,^59^ suggesting that glue-like traits may be more abundant than previously anticipated. Likewise, it has been shown that proteins have an intrinsic, evolved tendency to form complexes,^254^ with random mutations easily prompting the emergence of new interfaces. Thus, a suggested way forward for the design of MGDs is to elaborate derivatives of known ligands located inside or in the vicinity of a PPI interface, with the goal of contributing additional hotspots to the interface and increasing affinity for new protein interactors (Figure 5). For example, thalidomide and other IMiD-inspired derivatives have been used to design focused libraries of putative MGDs. These molecules are expected to bind the E3 CRBN ligase but are diverse in their capacity to recruit POIs.^255^

Similar design principles could be applied to ligands bound in the POI side, instead of the E3 ligase side. In this case, drug design notions borrowed from the discovery of PPI modulators could be implemented.^256,257^ In particular, detection of hotspots in flat hydrophobic (interface-like) regions of proteins, as well as fragment-based virtual screening methods, will provide initial hints to accommodate the seed ligand in the POI structure (Figure 5). Evolving these seed ligands into glues that recruit E3 ligases could be done by chemical library expansion strategies, or by rational modification of the solvent-exposed region of the seed molecule. Here, the goal would be to craft additional synthetic hotspots within the weak interaction interface, so that the surface can be recognized with sufficient affinity by the E3 ligase. Although no dedicated methods exist to achieve this goal, systematic analysis of residue–residue contacts observed in known PPIs, and the study of degrons,^258^ may offer insights into the necessary pharmacophoric features to be met. Recently, an optimization cycle guided by docking was able to evolve a promiscuous ligand of 14–3–3 proteins to a selective PPI inhibitor.^259^

Finally, MGDs may also function from an allosteric site.^260^ In this case, the small molecule does not participate directly in the interaction. Rather, it exposes or modifies a PPI interface by binding to a distant pocket that stabilizes a (non-native) conformation of the protein. Allostery has been suggested as a promising approach to modulating PPIs,^261^ since allosteric sites are more ligandable than PPI interfaces, in general. Key to discovering allosteric sites is to consider proteins as ensembles of structures, capturing feasible, but transient, conformational states.^262,263^ Translated to the problem of MGD discovery, and focusing on the POI, this involves identifying alternative conformations that reveal interesting PPI interfaces, followed by detection of allosteric cavities that would stabilize the given conformation in the presence of a ligand (Figure 5). While allostery may be a more far-fetched strategy to designing MGDs, there are several advantages in the approach that make it attractive from a computational viewpoint. First, the calculation can be thought of as a two-step procedure where the first step is independent of the small-molecule degrader. Given an E3 ligase, a large-scale docking run could be devised to screen each conformer of each POI independently. In a second step, satisfactory poses could be inspected to identify allosteric binding pockets and dock a ligand to those sites. While an approach like this one may be computationally intensive, it is easy to parallelize. Moreover, databases of structural ensembles of proteins exist^264^ and, even though in its by-default form AlphaFold2 was not optimized to sample conformations beyond the native structure,^265^ follow-up studies suggest that the tool has potential for generation of biologically relevant ensembles,^266^ although extensive validation beyond canonical examples and benchmarking against molecular dynamics simulations are still lacking. Systematic annotation of putative allosteric binding cavities is also possible, and has already been attempted on a proteome-wide scale.^267^

Overall, computational methods to enable prospective and rational design of degraders are still scarce. The relatively small number of known PROTACs and MGDs enforces that design is done primarily with a structure-based approach, which is particularly challenging due the complexity of assembling a ternary complex *de novo*. Supervised AI/ML methods are likely to become more relevant as more instances of small-molecule degraders are discovered. In this line, the aforementioned screening assays, as well as scalable methods to discover drug-induced PPIs such as Y2H-based/related assays,^268^ may generate the necessary data to include QSAR modeling in the loop of design. Recent modeling attempts have been done to predict half maximal (DC_50_) and maximal degradation (*D*_max_) concentrations of novel PROTACs.^269^ In the future, protein–ligand and PPI prediction models will have to be evolved to quantify their transient nature and eventually be incorporated in kinetic models for ternary complex formation.^270^ Despite its relevance to resolve complete and incomplete degradation behaviors, this remains a long-term goal.

## 5 Outlook

In this perspective, we have attempted to illustrate the status quo of how -omics technologies interface with AI/ML in order to advance research in TPD. We admittedly cover a lot of ground, aiming to review the relevance for proteomics-empowered, as well as AI/ML-enabled ligand discovery, and how they will synergize and complement each other. We have reflected on the successful history of phenotypic screens in the identification of MGDs and tried to illustrate how -omics and advanced imaging platforms will further diversify these approaches to enable researchers to unlock novel target classes. Importantly, we anticipate that AI/ML will play a key role in maximizing the actionable information content of these screens, possibly allowing the deduction of generalizable rules. Lastly, we aimed to reflect on the big challenge of prospective degrader design. While this is becoming a reality for the design of PROTACs, the *de novo* design of MGDs and the discussed approaches reflect, at this point, arguably rather a wish list that will need to be put into practice over the next couple of years. Success of these efforts will, among other factors, determine the overall influence of AI/ML for the TPD field.

What are other success factors? AI/ML is becoming a core component of drug discovery. Arguably, though, adoption of AI/ML methods in this field has been relatively slow and often received with skepticism, compared to other industrial applications.^271^ One reason for this is the fact that, as standalone tools, most AI/ML methods do not offer sufficient evidence to support their predictions. Rather, they are presented as “black boxes” that ingest an input (e.g., a molecule structure) and produce an output (e.g., a binding affinity prediction), without providing physical or mechanistic insights. Making more transparent AI/ML models, for instance by highlighting the structural moieties that determine a binding event, has been identified as a key factor to gain trust in AI/ML.^272^ Accordingly, “explainable” AI/ML benchmarks are flourishing, aimed at finding substructures in a molecule that determine bioactivity, in agreement with medicinal chemistry expertise.^273^ Modern algorithms such as “transformer models”, which have found tremendous success in the field of natural language processing and computer vision, have been applied to identify relevant atoms in a ligand or to pinpoint important residues in a protein binding site.^274^ Similarly, statistical methods to quantify contribution of certain chemical features to the predicted outcome have been suggested, borrowing concepts from “game theory”.^275^ In addition, current efforts are focused on identifying “causal” links between the input and the output of an AI/ML model. This often reveals punctual modifications of the input that cause abrupt changes in the output (a.k.a. “counterfactuals”),^276^ which should aid the rational design of small-molecules and the interpretation of -omics experiments, where many statistical correlations are discovered but only a few may have a mechanistic impact on the output.

Another limitation of most AI/ML models is that they do not assign a confidence interval to the predictions.^277,278^ Naturally, AI/ML models will be more confident if the input small molecule belongs to the same chemical space that was used at training time, while predictions will be less reliable if the input has completely new characteristics previously unseen by the model. The same applies to e.g. transcriptomics profiles of cell lines.^279^ Confidence estimation has been shown to be crucial to select true hits in virtual screening experiments, where a large proportion of molecules can fall outside the applicability domain of the AI/ML model. Indeed, in an AI/ML study based on kinase profiling data, considering confidence intervals remarkably increased the relevance of selected virtual hits.^280^ Likewise, AlphaFold2 provides a per-residue confidence measure that allows researchers to judge the quality of the predicted structure.^281^

The rapid adoption of AlphaFold2 can only be explained by unprecedented postpublication efforts to make the technology accessible to the broad scientific community. In fact, precalculated structures for 200 million protein sequences are already available online,^282^ which effectively removes any technical barrier to adoption. Unfortunately, though, the majority of existing AI/ML models remain in specialized journals, with poorly maintained code and variable rigor in validation.^283^ Initiatives like the DREAM Challenges,^284^ MoleculeNet,^110^ and Therapeutic Data Commons^285^ offer benchmark data sets across a wide spectrum of prediction tasks for pharmacology. With the emergence of user-friendly, readily available repositories of AI/ML models, it is likely that execution of AI/ML models will become routine practice in medicinal chemistry and chemical biology laboratories.

In conclusion, we anticipate a key role of AI/ML tools in answering central challenges in TPD research. In our opinion, the unique potential of AI/ML in this interdisciplinary context arises from three aspects. First, its capacity to learn numerical representations (embeddings) of small-molecules, proteins, and cellular phenotypes, which can then be unified in a single analytical framework. Second, its ability to decode these embeddings, yielding scalable generative models that can aid rational design. Third, its scalability in a virtual screening framework, holding promise for systematic discovery of hits across multiple proteins, binding sites, and conformations. Lowering the aforementioned barriers to adoption of AI/ML will be key to ensure an efficient wet-lab/dry-lab cycle and thus an important contribution of AI/ML in the progress of TPD and other proximity-inducing modalities.
